# Sertoli cell tumor in familial adenomatous polyposis: a case report

**DOI:** 10.3389/fonc.2026.1856272

**Published:** 2026-08-24

**Authors:** Chandra Kakarala, Katie Bussing, Reema Patel, Jessica Moss, Zin W. Myint

**Affiliations:** 1 Department of Internal Medicine, University of Kentucky, Lexington, KY, United States; 2 Department of Pathology, University of Kentucky, Lexington, KY, United States; 3 Markey Cancer Center, University of Kentucky, Lexington, KY, United States

**Keywords:** familial adenomatous polyposis, Sertoli cell tumor, sex cord-stromal tumor of the testis, testicular tumor, Wnt pathway

## Abstract

Familial adenomatous polyposis is known to progress into colorectal cancer, and extra-intestinal malignancies have also been reported. However, testicular sex cord stromal tumors are very rarely noted in association. In this study, we report the case of a 50-year-old man with genetically confirmed FAP and adenocarcinoma of the colon who was incidentally found to have a testicular lesion. Radial orchiectomy was performed, and histopathologic examination confirmed a Sertoli cell tumor without high-risk features. This tumor was found to have diffuse expression of nuclear beta-catenin, a marker for Wnt pathway activation, suggesting an abnormal CTNNB1 or APC variant. This suggests a possible link between germline APC mutations and Sertoli cell tumor development.

## Introduction

Triggered by a germline loss-of-function mutation in the adenomatous polyposis coli (APC) gene, familial adenomatous polyposis (FAP) leads to the development of hundreds of colorectal adenomatous polyps. This confers a near 100% risk of progressing into colorectal cancer (CRC) in the absence of a prophylactic colectomy (1, 2). Commonly seen extra-intestinal manifestations of FAP include osteomas, dental abnormalities, papillary thyroid cancer, benign skin tumors, hepatoblastomas, malignancies of the pancreas and biliary duct, medulloblastomas and other central nervous system tumors (3). Testicular sex cord stromal tumors (TSCST) have only rarely been reported to be associated with FAP (4).

## Case description

A 50-year-old man with a 30-pack-year smoking history presented with a month of fatigue, abdominal pain, and alternating constipation and diarrhea. He also had an unintentional weight loss of 18 kg over the preceding three months. He denied scrotal pain, swelling, dysuria, or nocturia. He had no prior abdominal surgeries or colonoscopies. Family history was significant for FAP and prostate cancer in his grandfather. On presentation, he was afebrile and mildly hypertensive (blood pressure 155/96 mm Hg), a pulse of 63 beats per minute, and an oxygen saturation of 100% on room air. On physical examination, he was diffusely tender over the abdomen. Testicles were bilaterally descended with a 1.5–2 cm non-tender solid mass noted along the posterior superior margin of the right testicle. There was no gynecomastia. His Eastern Cooperative Oncology Group (ECOG) performance status was 1.

**Figure 1:**
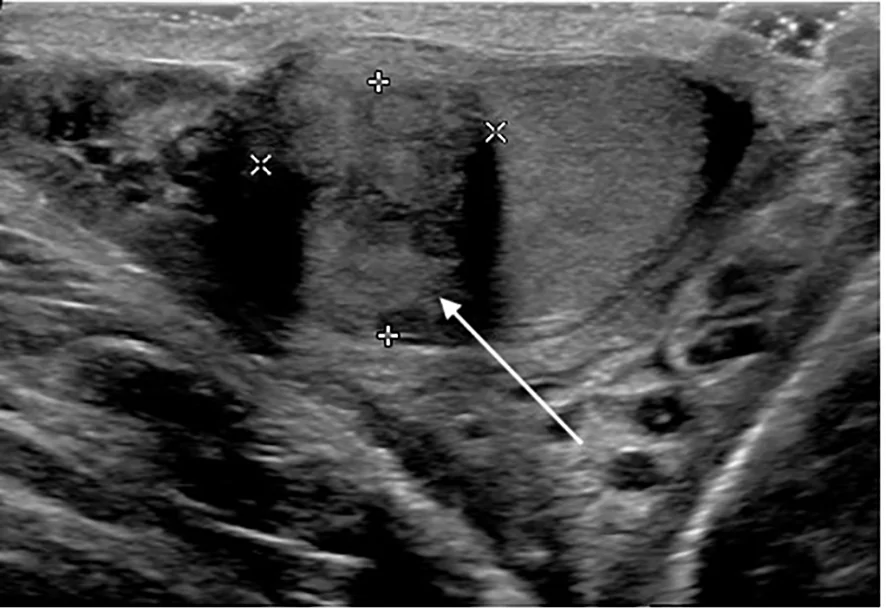
Gray-scale scrotal ultrasonography image, with the white arrow indicating an approximately 1.7 x 1.6 x 1.4 cm solid lesion in the superior pole of the right testis.

**Figure 2:**
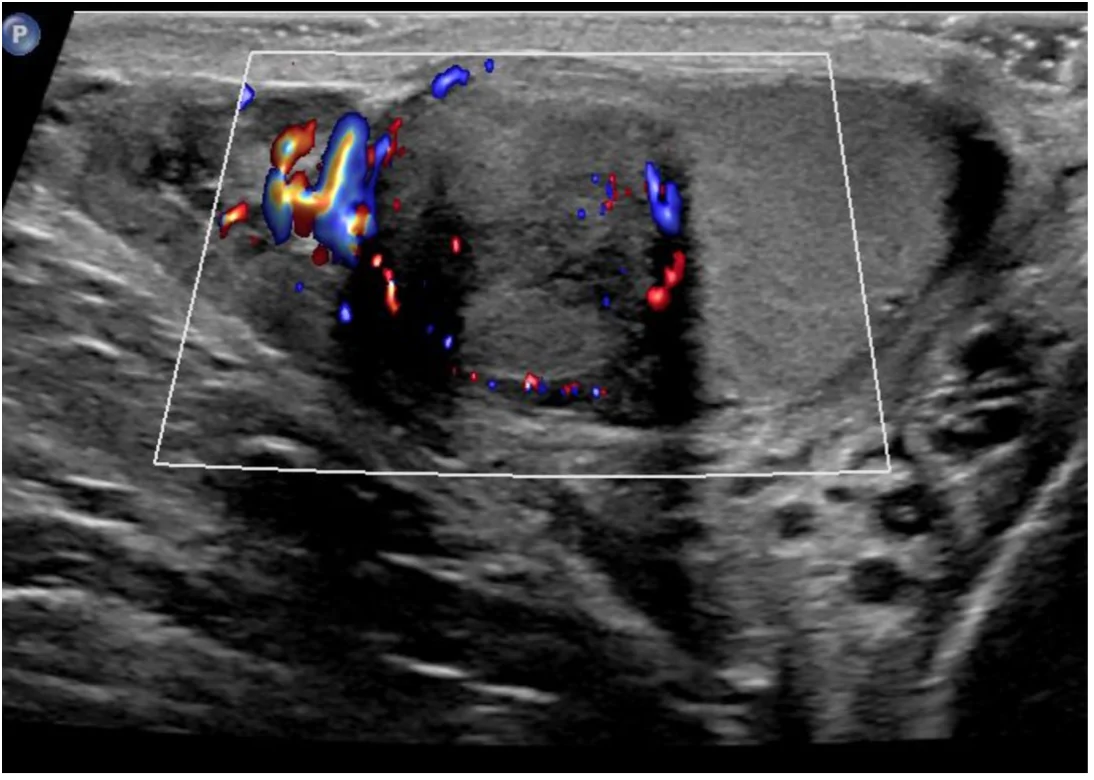
Color Doppler evaluation demonstrating internal vascularity, consistent with a vascular intratesticular lesion.

Two months later, he underwent a colonoscopy, which was notable for diffuse polyposis in the entire colon and a cecal mass. Genetic testing revealed a heterozygous pathogenic mutation c.3780_3781delGA (p.Q1260Hfs*15) in the APC gene. A computed tomography (CT) scan of the abdomen and pelvis was significant for a 2 cm high-density focus on the right testicle, and follow-up scrotal ultrasound revealed a 1.7 x 1.6 x 1.4 cm isoechoic mass in the superior pole of the right testis with internal vascularity suspicious for a primary testicular neoplasm (**Figures 1**, **2**). Initial CEA was elevated to 10 ng/mL, but other tumor markers were within the normal range. AFP was <2.3 ng/mL, LDH 238 U/L and hCG <1 mIU/mL.

He underwent a laparoscopic total colectomy six months after first presentation. Histopathology was notable for a pT2, pN1a adenocarcinoma with mucinous features and multiple adenomas with focal high-grade dysplasia. Immunohistochemistry testing for mismatch repair proteins was negative. Two months later, he underwent a right orchiectomy, which was preferred over testis-sparing surgery as preoperative imaging could not definitively rule out malignancy, and radical orchiectomy remains the standard of care when the contralateral testis is normal. Initial circulating tumor DNA (Signatera) was obtained for baseline minimal residual disease assessment and found to be negative.

**Figure 3:**
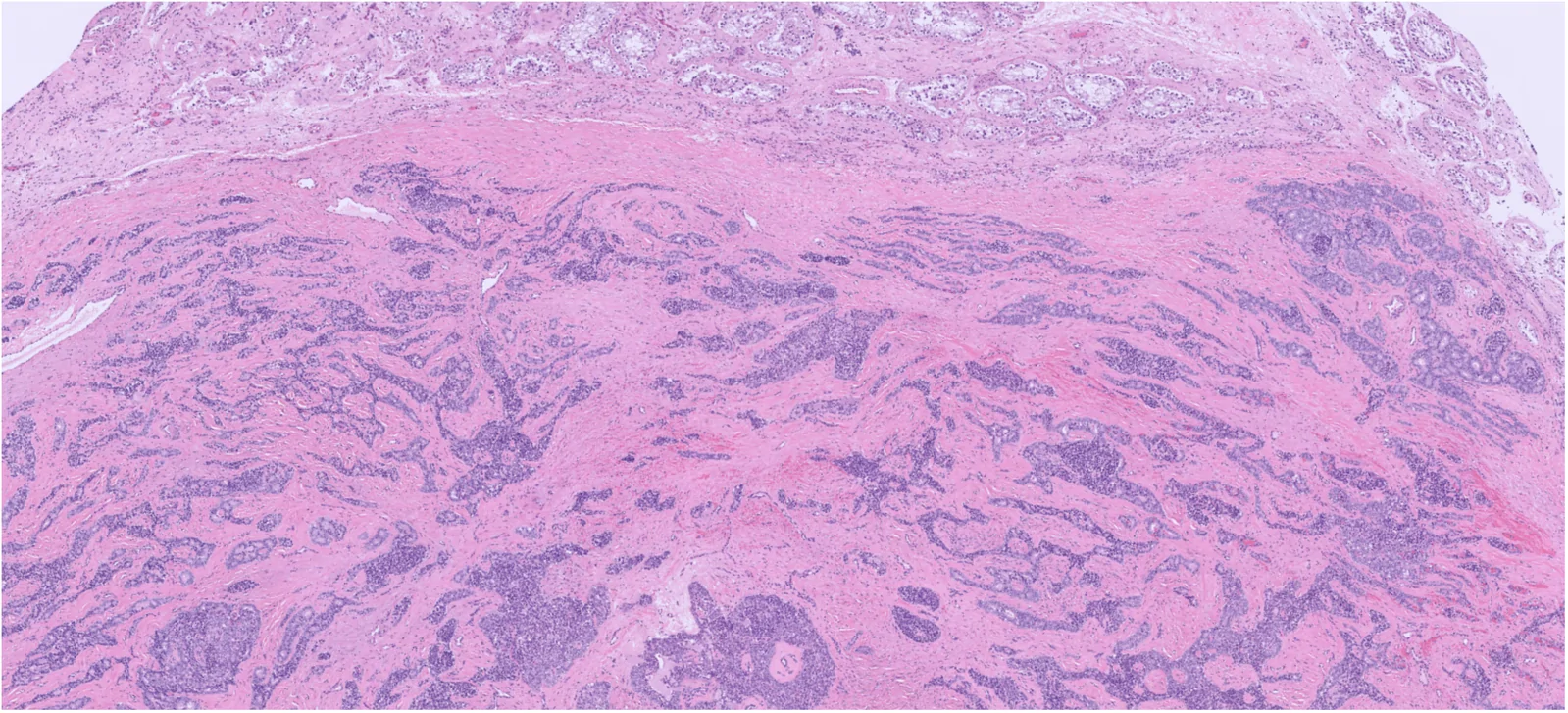
On low power examination of the patient’s SCT, the tumor is well-circumscribed, with a distinct border between the normal seminiferous tubules (top) and the tumor (bottom). Tumor cells are arranged in round and elongated tubules, with frequent tubular fusion and occasional cord-like growth. The surrounding stroma is dense, fibrovascular and sclerotic. Lymphovascular invasion and extra-testicular extension are not identified. Hematoxylin and eosin (H&E) staining; original magnification x2.

**Figure 4:**
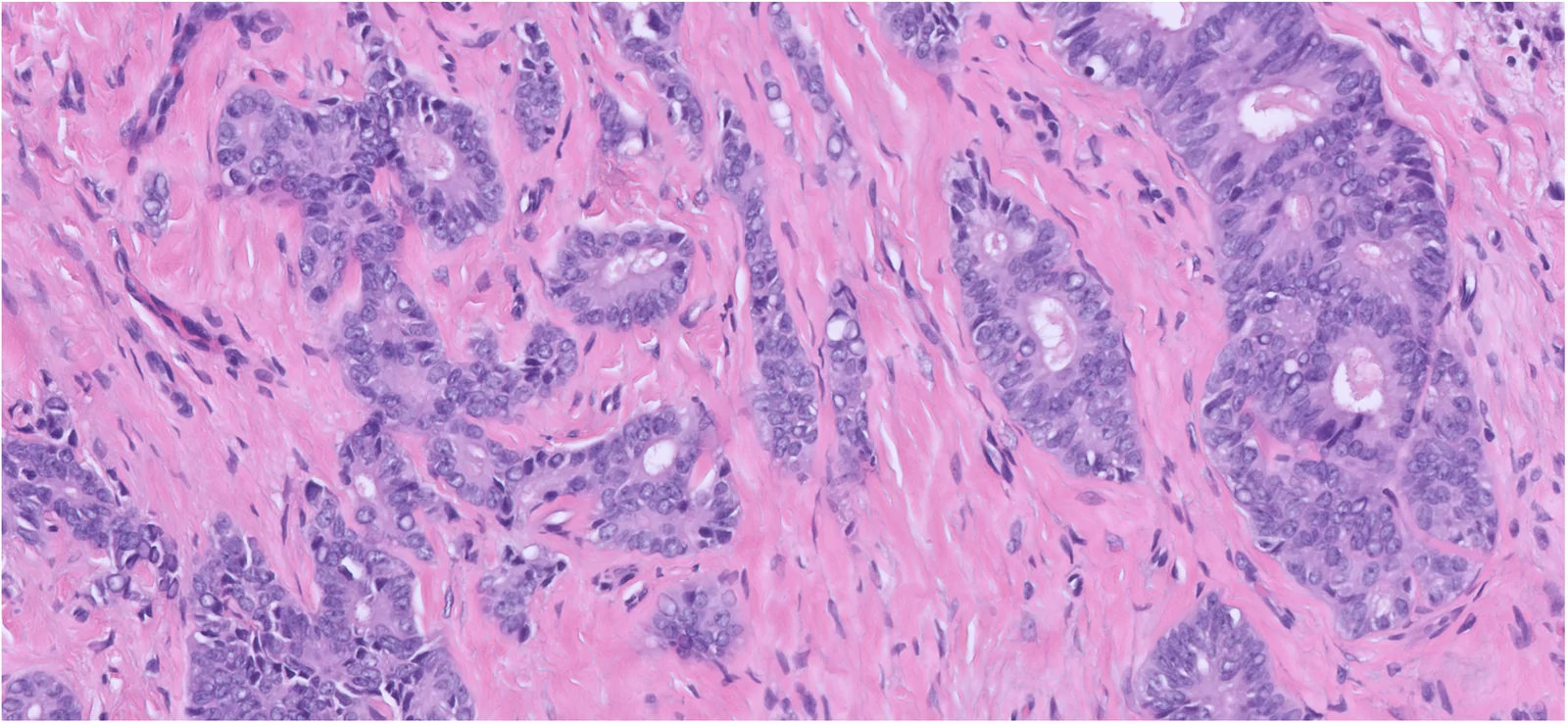
On high power examination of the patient’s SCT, tubules are lined by columnar cells with a moderate amount of lightly eosinophilic cytoplasm. Nuclei are monomorphic and round to oval, with occasional pinpoint nucleoli and nuclear clearing. Necrosis, nuclear pleomorphism, and significant mitotic activity are not identified. Hematoxylin and eosin (H&E) staining; original magnification x20.

**Figure 5:**
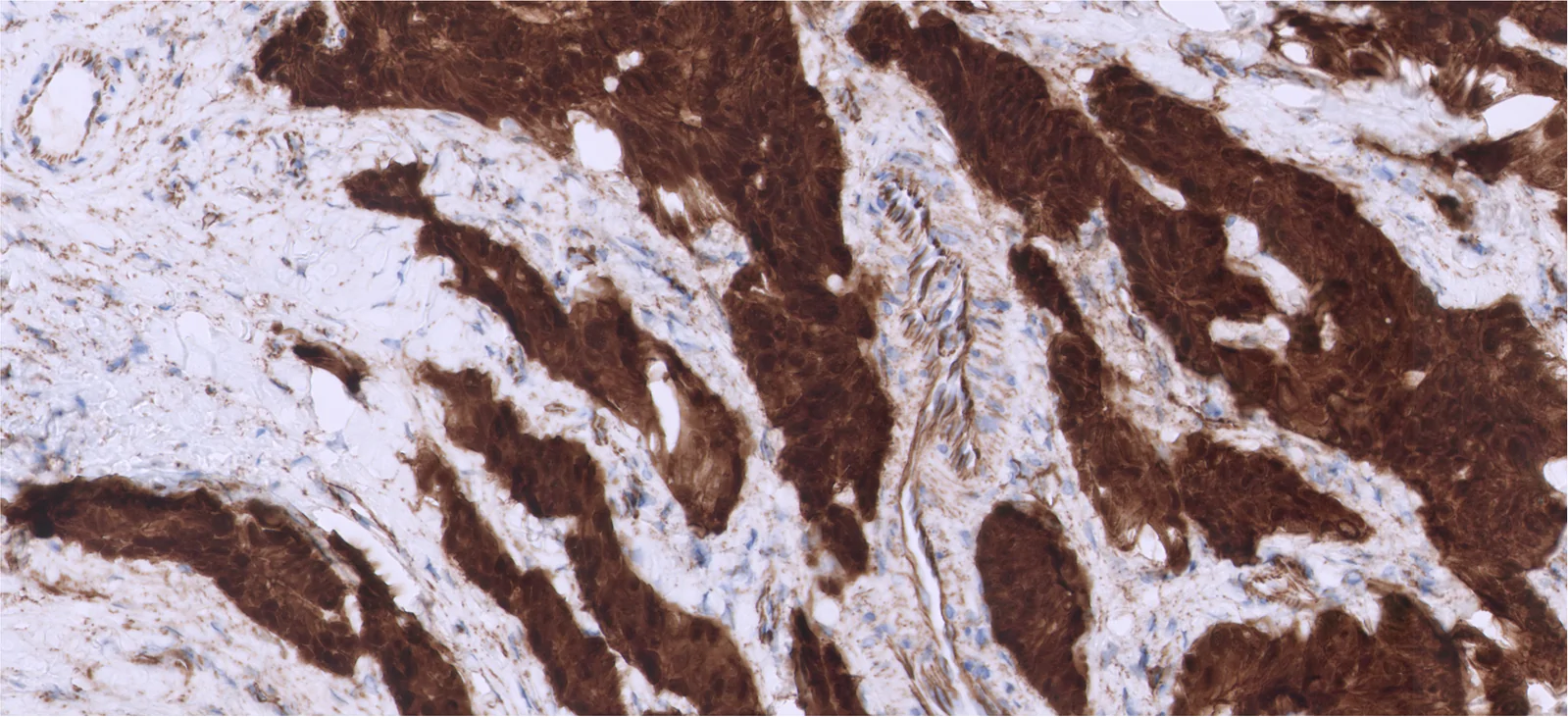
Immunohistochemical staining for beta-catenin of the SCT shows diffuse, strong nuclear and cytoplasmic expression. The presence of nuclear beta-catenin expression is aberrant and supports activation of the Wnt pathway. Original magnification x20.

A broad differential was considered for the testicular neoplasm. The lesion’s small size and normal tumor markers favored a primary testicular neoplasm and was suggestive of a seminoma or a non-germ cell neoplasm. Non-germ cell tumors such as Sertoli cell tumor (SCT) and Leydig cell tumor were higher on the differential due to his age. Pathology reports noted a 1.8 cm pT1a SCT and no high-risk features (**Figures 3**, **4**). Immunohistochemistry was notable for diffuse strong cytoplasmic and nuclear expression of beta catenin (**Figure 5**). CTNNB1 sequencing of the mass was not performed, but SF-1 was patchy positive.

**Figure 6:**
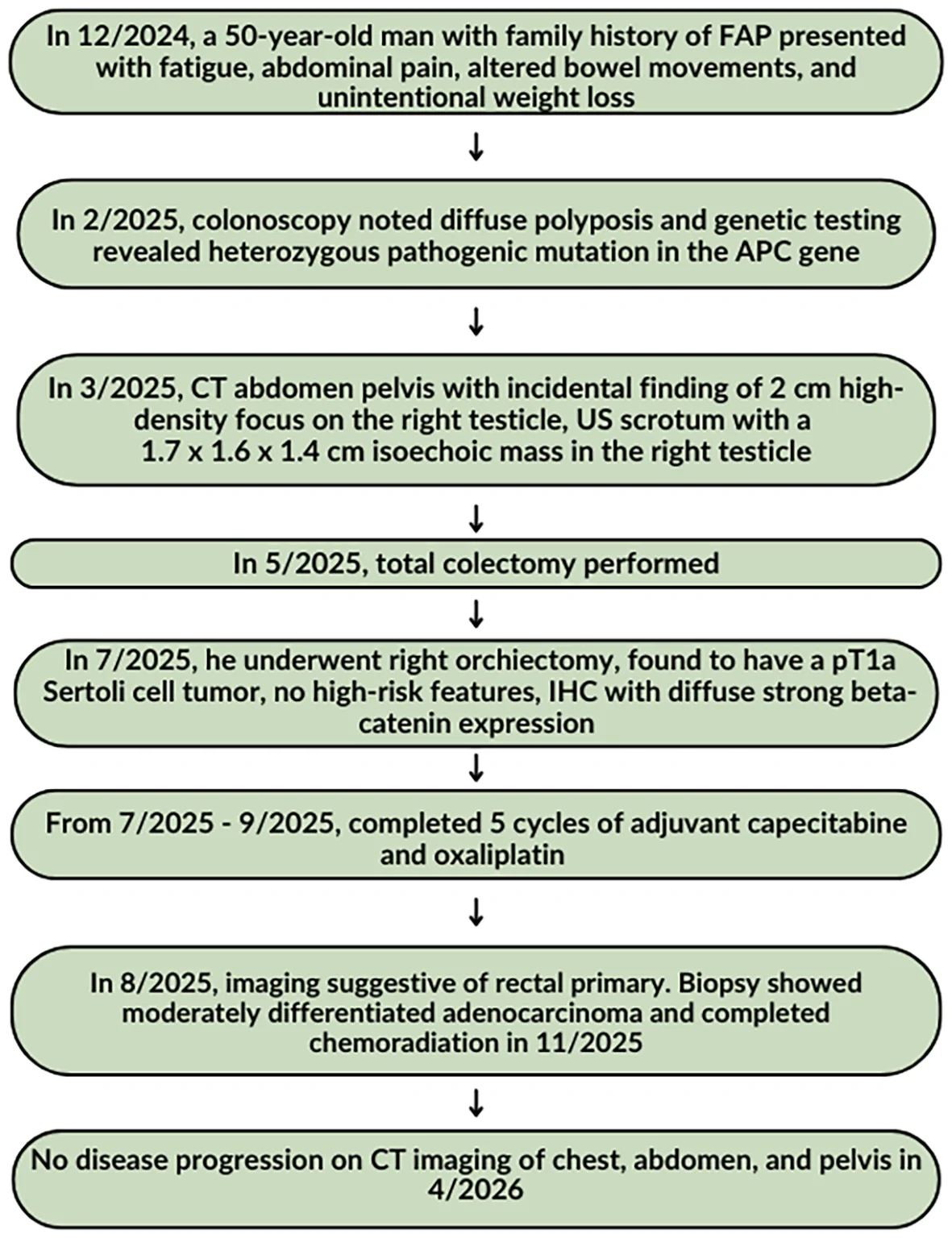
Timeline of symptoms and relevant imaging with interventions (FAP, familial adenomatous polyposis; APC, adenomatous polyposis coli; CT, computed tomography; US, ultrasound; IHC, immunohistochemistry).

Adjuvant chemotherapy with capecitabine and oxaliplatin was initiated two months after colectomy. Chemotherapy was overall well tolerated for five total cycles, apart from requiring a 20% dose reduction in oxaliplatin due to chemotherapy-induced neuropathy. Soon after starting chemotherapy, imaging of his abdomen and pelvis was concerning for a rectal primary. Flexible sigmoidoscopy was performed and he was found to have moderately differentiated adenocarcinoma of the rectum and was started on neoadjuvant chemoradiation therapy followed by abdominoperineal resection. No evidence of disease progression was noted in CT imaging of chest, abdomen, and pelvis in April 2026. A timeline of symptoms is illustrated in **Figure 6**.

Written informed consent was obtained from the patient for the publication.

## Discussion

Testicular tumors are uncommon, accounting for 1-2% of all neoplasms in men (2). Given the rarity of SCTs and the limited clinical guidance available, we provide a comprehensive narrative review of their epidemiology, pathologic risk factors, molecular features, and management considerations relevant to medical oncologists. Testicular sex cord tumors (TSCTs) comprise about 4% of all testicular tumors and have significant heterogeneity in morphology, immunohistochemistry and genetic associations (1, 5). The second most common type of TSCTs are SCTs, which make up 1% of all testicular tumors (4). SCTs are classified, in decreasing order of prevalence, into “not otherwise specified” (NOS), intratubular large cell hyalinizing SCTs, and intratubular large cell calcifying SCTs (1, 5).

A large meta-analysis of 435 patients with SCTs described the median age at diagnosis to be 29 years. The most common initial manifestation was a testicular mass (58%), followed by gynecomastia (15%), and scrotal pain in 11% (1). SCTs are described as largely unilateral, typically between 3 to 4 cm, well-circumscribed tan, grey or white tissue with occasional areas of hemorrhage and cystic components, sometimes resembling seminomas (6). Microscopic evaluation is highly variable, ranging from intricate tubular differentiation, often in a nodular pattern separated by acellular or vascular stroma, to absent tubular differentiation (6). Nuclear expression of SF1 has been noted consistently in SCTs and is used to classify a tumor as sex cord stromal in origin (7, 8). Additionally, 60-70% of SCTs NOS were found to have a CTNNB1 gene mutation with nuclear expression of B-catenin on immunohistochemistry (8, 9). While LEF1 is an emerging surrogate marker of Wnt pathway activation, the morphologic and immunophenotypic features, including diffuse nuclear and cytoplasmic beta-catenin positivity, are supportive of the diagnosis in this case. LEF1 immunohistochemistry was not present at our institution at the time of this case report (10). These features underscore the broad clinicopathologic spectrum of SCTs and provide important context of subsequent discussions on malignant risk stratification and treatment approaches.

Metastatic disease is relatively infrequent, seen in around 11% of patients. It involves, in decreasing order of frequency, the retroperitoneal lymph nodes (RPLNs), lungs, bones, inguinal lymph nodes, liver, brain and kidneys (1). The 5^th^ edition of the World Health Organization (WHO) classification of testicular tumors identifies necrosis, vascular invasion, nuclear pleomorphism, increased mitotic activity, and high-grade cytologic atypia as features associated with malignant behavior (5). While the 4^th^ edition recognized tumor size > 5cm as an adverse prognostic factor for metastases, a meta-analysis by Grogg et al. suggests that a lower tumor size threshold of 24 mm may better predict metastatic potential. Spermatic cord invasion has also been identified as an adverse feature (1, 11). Significant differences have also been identified in the molecular framework of metastasizing and non- metastasizing SCTs. Metastasizing SCTs were shown to harbor heterogenous mutations in genes associated with the TP53 pathway, cell cycle regulation, and maintenance of telomere length (12). They also noted that non-metastasizing SCTs were driven exclusively by the activation of the Wnt pathway, while metastasizing SCTs often had other alterations (12). At present, these molecular findings provide biologic insight into tumor pathogenesis but do not directly inform therapeutic decision-making or surveillance strategies.

Although most patients with SCTs are initially managed surgically, medical oncologists are frequently consulted regarding the role of systemic therapy, lymph node dissection, and surveillance, particularly in cases with high-risk features or metastatic disease. For patients with 1 or no high-risk pathological features, partial or total orchiectomy remains the most effective initial approach (8). The rate of local and contralateral recurrence in patients with partial orchiectomies, or testis sparing surgery (TSS), is very low, estimated by one meta-analysis to be <1% (1). Infrequently, additional treatment with an aromatase inhibitor, such as testolactone or anastrazole, is initiated to reduce gynecomastia (1). It is unclear whether RPLN dissection (RPLND) has any effect on survival or future recurrence. It may be favored immediately after orchiectomy in patients with small-volume metastasis, especially when limited to retroperitoneal disease, and tumors with two or more high risk features (13, 14). For these patients, treatment at the time of diagnosis is further supported by studies that have demonstrated delaying treatment to be linked to worse outcomes, such as higher volume disease, greater relapse rates and treatment failure (14). No standard chemotherapeutic regimen has been established, with traditional chemotherapies largely noted as ineffective.

Guidelines for follow-up are variable; however, it has been proposed that post-TSS patients with no risk factors for metastatic disease undergo follow-up with ultrasound surveillance of the testis and no RPLND, and patients with risk factors undergo follow-up with imaging of chest, abdomen, and pelvis (1, 14). Overall median survival of patients with metastatic disease was found to be poor, around 20 months (2).

The association of SCTs with systemic disease has long been explored. Patients with the Carney complex, an autosomal dominant genetic disorder, have been found to develop large cell calcifying SCTs, while patients with Peutz-Jeghers syndrome are associated with intratubular large cell hyalinizing SCTs (15). However, the association between FAP and SCTs has only rarely been reported in the literature (see **Table 1**) (16–18).

**Table 1:** Summarizes previously published occurrences of FAP and SCTs.

Demographics and presentation	Pathological and molecular findings	Management	Outcome	References
34-year-old male with history of FAP with subtotal colectomy at 15	1.4 cm and 1.3 cm Sertoli cell tumors, strong nuclear reactivity with beta catenin	Bilateral orchiectomy	No long-term follow-up, postoperative course uneventful	(16)
23-year-old male with FAP (APC germline mutation)	Sertoli cell tumor, malignant features and strong positive staining for β-catenin	Unknown	No long-term follow-up available	(17)
29-year-old with history of FAP and known APC variant	A typical Sertoli cell tumor, NOS and a purely intratubular small Sertoli cell neoplasm, diffuse nuclear beta-catenin expression	Unknown	No recurrence	(18)
38-year-old with clinical history of FAP but unknown molecular status	Sertoli cell tumor NOS, diffuse nuclear beta-catenin expression	Unknown	No recurrence	(18)
23-year-old with unknown clinical history of FAP, but known APC variant	Sertoli cell tumor NOS, diffuse nuclear beta-catenin expression	Unknown	No recurrence	(18)
50-year-old with FAP (APC germline mutation)	1.8 cm Sertoli cell tumor, NOS, diffuse strong cytoplasmic and nuclear expression of beta catenin	Orchiectomy	No recurrence till date	Present case

Affecting 1–8 per 1000 people, FAP is an autosomal dominant disease leading to the development of thousands of adenomatous polyps, and eventual development of CRC in the absence of a colectomy (19, 20). Bilateral SCTs in a patient with history of FAP were first described in 2012 by Xiao et al. (16).

**Table 1**. Demographics, pathological and molecular Findings, management and outcomes of SCTs associated with FAP.

The Wnt signaling pathway, a key driver in both FAP and SCTs, is well-established to be fundamental in growth and development control (20, 21). It is also known as a driver of carcinogenesis by activating genes involved in cell proliferation, survival and maintenance (20). The cytoplasmic protein β-catenin is a critical component of this pathway and functions as a transcriptional licensing factor (20, 21). As the Wnt pathway is activated, β-catenin proliferates in the cytoplasm and nucleus, where it interacts with T-cell factors (TCF) transcription factors, activating genes controlling cell proliferation (21, 22). β-catenin is encoded by the gene CTNNB1, and its stability is regulated by the destruction complex (DC), comprising of a scaffold protein Axin, the tumor suppressor protein APC, and two serine-threonine kinases (21).

FAP is well-known to be associated with a germline loss-of-function mutation in a single allele of APC, after which a second, somatic inactivation of the remaining APC allele in the colon leads to loss of heterozygosity (LOH) (20). APC loss impairs DC function and increases β-catenin levels, thus causing perpetual cell regeneration and tumorigenesis (21). Interestingly, studies investigating other extracolonic malignancies in FAP have noted biallelic APC gene inactivation, such as in desmoid tumors and hepatoblastoma (23, 24).

Meanwhile in SCTs, gain of function CTNNB1 or inactivating APC variants were seen at a combined frequency of >90% in non-metastasizing SCTs (11). Notably, immunohistochemistry was proven to be an exemplary marker for genomic alterations, as all tumors with pathogenic CTNNB1 or APC variants also portrayed diffuse nuclear β-catenin expression (12). More recently, Siegmund et al. noted that TSCTs in patients with pathogenic germline APC variants lacked gain-of-function CTNNB1 mutations, thereby strengthening the claim that development of TSCT was due to subsequent APC inactivation leading to LOH (18). This was bolstered by comparative studies of non-neoplastic and lesional tissue in their cases, which suggested germline APC variants and LOH to be the oncogenic driver of the SCT. Our tumor was not sequenced for CTNNB1, which is a limitation of our case study.

While only very few SCTs have been reported in the literature in patients with FAP, all have been reported to have diffuse expression of nuclear beta-catenin, a marker for Wnt pathway activation, which suggests an abnormal CTNNB1 or APC variant. Although the number of reported cases remains limited, these observations suggest a possible association between germline APC mutations and the development of SCTs. This relationship remains hypothesis-generating and warrants further investigation in larger cohorts.

## Data Availability

The original contributions presented in the study are included in the article/supplementary material. Further inquiries can be directed to the corresponding author.
